# Phonemic–Phonological Profile of People with 22q11.2 Deletion Syndrome: A Pilot Study

**DOI:** 10.3390/brainsci15030298

**Published:** 2025-03-12

**Authors:** Esther Moraleda-Sepúlveda, María Rubio-Lorca, Noelia Pulido-García, Noelia Santos-Muriel, Javiera Espinosa-Villarroel

**Affiliations:** 1 Facultad de Psicología y Logopedia, Universidad Complutense de Madrid, 28040 Madrid, Spainjavierae@ucm.es (J.E.-V.); 2 Facultad de Ciencias de la Salud, Universidad de Castilla-La Mancha, 45600 Talavera de la Reina, Spain; mariarubiolorca@gmail.com

**Keywords:** 22q11.2 deletion syndrome, language, speech, phoneme adquisition

## Abstract

**Background:** 22q11.2 deletion syndrome is considered as a rare disease. It is considered one of the most prevalent genetic disorders with multiple systemic and neuropsychological alterations. At present, there are few studies that define the linguistic profile in Spanish of children with this syndrome. **Objectives:** Therefore, the aim of the present study was to define the phonemic–phonological characteristics of people with 22q11.2 Syndrome. **Method:** Eight boys and girls between 5 and 16 years old participated in an evaluation using the following tests: Induced Phonological Register and Laura Bosh’s Phonological Assessment and Children’s Speech. **Results:** After analyzing the results obtained, it was observed that more than half of the participants presented a delay in the acquisition of phonemes. **Conclusions:** The conclusion of this study points out the importance of working on language, especially the phonetic-phonological area, throughout the development of people with 22q11.2 Syndrome.

## 1. Introduction

22q11.2 deletion syndrome is an autosomal dominant disorder characterized by the deletion of a segment in region 11.2 of chromosome 22. It can cause decreased muscle tone; delayed growth, development, and learning; frequent respiratory infections; and behavioral disorders, among other problems [1,2,3,4].

It is currently considered a rare disease since its frequency is less than 5 cases per 10,000 inhabitants. It has an incidence of 1 per 5950 newborns [5]. The prevalence of the syndrome described so far varies from 1 in 4000 [6] to 1 in 6395 individuals born [7]. However, due to clinical variability, it is likely to be higher since many cases go unnoticed due to low clinical expressiveness [8]. Even so, it is the most frequent genetic cause of cleft palate [9] and also the second most frequent cause of developmental delay after Down syndrome [10].

Some of the most common facial phenotypic characteristics are looped or dysmorphic ears, oral respirator facies, hypotonic cheeks, parted lips, prominent nose, wide and bulbous tip with nostril hypoplasia, and lowered corners of the lips [2]. Along the same lines, cleft lip and palate, hypertelorism, enamel hypoplasia, dental agenesis, delayed dental eruption, and hypodontia and micrognathia are observed [11,12,13]. It is estimated that there are structural alterations of the palate in 90% of cases [14]. Palate abnormalities can impair speech, voice, and pronunciation [15,16]. These alterations, together or individually, can cause velopharyngeal insufficiency, which has been reported in 70% of people with 22q11.2 [17]. However, the 22q11.2 phenotype is quite variable, and there may be marked differences between those affected [10].

As regards hearing, hearing loss and recurrent otitis media are often observed [10]. The most common hearing loss is conductive and is related to chronic episodes of otitis media. However, 15% of cases involve some degree of sensorineural hearing loss, the majority being unilateral and mild [18]. As a consequence, the discrimination of phonemes during speech can be affected considerably impairing their acquisition and production [19,20].

At the cognitive level, cases vary from normal intellectual capacity to functional diversity of a moderate intellectual type. Approximately 50% of people typically score with an IQ below 70 [10]. Therefore, cases of generally mild intellectual disability, borderline or normal cognitive ability, and/or learning disorders may be referred to [21]. All this means that people with 22q11.2 syndrome exhibit a characteristic psychoeducational profile. By school age, most children have impaired reading comprehension, math, and problem-solving [22,23]. In contrast, they are stronger in the areas of decoding in reading, spelling, and phonological skills [17,23].

### Speech and Language Characteristics in People with 22q11.2 Syndrome

With regard to language, developmental delay is described in the vast majority of people, being close to 90% [10,24]. In fact, this delay is one of the main characteristics of people with 22q11.2 deletion syndrome. The first words are usually not spoken until 24 months, and in some cases, this occurs later [25]. Moreover, 90% of these children do not use oral language at two years of age, 80% do not use it at three, and 30% do not at four [26]. These difficulties in language development continue between 6 and 16 years of age, with significant differences in all areas of language compared to their typically developing peers [27].

Some authors conclude that people with 22q11.2 deletion syndrome experience greater alterations in receptive language than in expressive language [28,29,30,31]. At the productive level, the four components of language are affected: phonetics, lexicon, syntax, morphology, and pragmatics [32,33]. They exhibit alterations in vocabulary, difficulties in learning and accessing new words, and problems following orders of various slogans. Difficulties are also observed in talking about everyday life, conveying ideas, thoughts, and feelings, as well as listening, asking for help, and participating in conversations [2,30,33,34].

It should be taken into account that sometimes language difficulties tend to turn into pragmatic alterations that interfere with social interactions, altering social communication [35,36]. They carry out irrelevant communicative acts in certain contexts and provide little information [37]. If we take gender into account, girls have better pragmatic skills [30].

Specifically, in the areas of speech and phonemic–phonological difficulties, more than half of children with 22q11.2 deletion syndrome have serious problems in the articulation of several phonemes [38]. The aforementioned hypernasality decreases speech intelligibility [2]. As indicated by [39], phonological development culminates at the age of 7, although a percentage of children will not yet have finished acquiring all the phonemes. Children with this syndrome follow the same typical developmental pattern but with a later onset and a longer generalization time [30,40]. As a general rule, they use compensatory articulatory patterns and the glottal stop /ʔ/ to replace the production of oral and nasal consonant sounds [38,40,41].

Thus, they experience greater difficulties in the production of late-acquired phonemes, such as fricatives, vibrant rhotics, stops, affricates, liquids, nasals, and consonant clusters /r/, /l/ [30,40,41,42]. However, speech articulation disturbances and poor intelligibility improve significantly with age [43].

Articulatory precision is diminished and imprecise, and the articulation points of each of the phonemes are not marked correctly, causing intelligibility problems in numerous situations [30]. The research focused on Spanish-speaking children considers that the youngest participants with 22q11.2 syndrome had a delay in the acquisition of phonemes (except nasal consonants) compared to their normative group, and the majority made use of the compensatory sound. Participants older than 8 years correctly produced late-acquired phonemes, although specific articulation difficulties persisted [44].

Speech sound production skills have been shown to be worse in children with 22qDS compared to children with isolated cleft palate [42,45,46,47] or Trisomy 21 [48], suggesting that the presence of velopharyngeal and palatal insufficiency or developmental delay alone does not account for the severity of the speech deficits in 22qDS. It seems, therefore, proven that people with 22q11.2 Syndrome show significant risks of developing speech sound disorders [49].

Studies relating to the level of speech and language in 22q11.2 Syndrome are quite limited [50]. Gerdes et al. (1999) [51] found no difference between children with 22q11DS with and without palatal abnormalities on standardized language outcomes. Another example is the studies by Solot et al. (2001) [26], who found no correlation between language, speech, and palatal anomalies in their sample of school-age children with 22q11.2 Syndrome. These results seem to suggest that palatal anomalies do not directly influence language development. However, other studies suggest that the deterioration of language development also specifically affects the area of phonetics and phonology [52,53,54].

However, there is little research in this regard since it is considered a rare disease, and there is not sufficient research concerned with the speech sound skills of Spanish-speaking children with 22q11.2 deletion syndrome. Therefore, the goal of this study is to determine, analyze, investigate, and describe the phonemic–phonological profile of people with 22q11.2 deletion syndrome. The central hypothesis of this research is that people with 22q11.2 syndrome present a different phonemic and phonological pattern in their acquisition. The justification for this pilot study has its main origin in the scarcity that covers the phonemic–phonological component in Spanish, and it is essential to determine these characteristics in order to propose an adequate speech therapy intervention.

## 2. Materials and Methods

### 2.1. Participants

The sample is made up of a single group of eight voluntary participants of both sexes (50% men, 50% women), aged between 5 and 16 years (mean age: 9.8 years). The selection criteria to participate in the study were as follows: a confirmed genetic diagnosis of 22q11.2 deletion syndrome, the consent of the child’s mother/parent/guardian, residence in Spain and Spanish as the child’s mother tongue. All the participants present normal cognitive skills without the presence of other neurodevelopmental problems. Cognitive development had been previously evaluated by a psychologist and was one of the requirements for inclusion in this study. Parents had to provide clinical reports. The participants did not present anatomophysiological alterations other than those that are part of the syndrome itself. However, all of them need speech and language therapy as part of their special education services. The characteristics of the sample can be observed in Table 1.

### 2.2. Procedure

Initially, the 22q11.2 Syndrome Association of Madrid was contacted for orientation and informative purposes concerning the project. On the basis of this contact, the study sample was defined through a database. For this purpose, a document was submitted to the Association setting out the goals of the research and the purpose of the study. This entity contacted the families of patients with the syndrome and sent them a letter explaining what the study included. Once the families communicated their interest in participating in the project, the consent was sent and signed.

Previously, the study was accepted by the respective bioethics committee of the Faculty of Health Sciences of the University of Castilla-La Mancha. This study was approved in April 2021 by the ethics committee of the Clinical Research Ethics Committee of the Integrated Area of Talavera de la Reina, with number 49/2021.

Afterwards, first contact with the participants was made by email or telephone. They were informed of the research and the evaluations they would undergo. At the same time, the days and times of the sessions were arranged with each of the families, adapting to the availability of both the child and the tutor.

Later, the evaluations took place online through the Microsoft Teams platform, keeping ambient noise levels to a minimum and ensuring a good internet connection. One day before each session, the family received an email as a reminder with the pertinent information and the link to join the session on the scheduled day. The reason why the participants were evaluated online was because all the participants belonged to the 22q11.2 Spain association, so in most cases, it was impossible for the professional team to physically travel to their places of origin. The tests that were used were two: the Induced Phonological Register and the Laura Bosch Phonological Register. Attempts were made at all times to maintain naturalness and spontaneity. The children were evaluated in one day, in a single 45 min session, except for one participant who required the use of two 45 min sessions in order to be evaluated. All the responses were recorded in writing at the time of the evaluation, and once the session was over, the responses were scored by the principal investigator, a specialist in language development.

Finally, after carrying out the evaluations and collecting the data on the test record sheets, the analysis and results were obtained. They were interpreted using the manuals pertaining to these standardized tests.

An inter-judge evaluation was carried out between three evaluators to verify the results obtained. The responses were video recorded and the tests were later corrected. Three external evaluators were used to avoid possible response and interpretation biases. To compare the results between the judges, the Kappa Coefficient was used with the statistical program SPSS 29.0. The Kappa coefficient of agreement between the judges was 0.84.

Spearman’s Rho was employed to analyze the correlation between the variables of both tests. The results was 0.85, so indicating that the same articulation pattern existed in the participants, regardless of the test used for evaluation.

### 2.3. Instruments

To carry out this study, evaluation tests were selected to analyze the phonemic–phonological characteristics of Spanish-speaking boys and girls. On the one hand, the first test used was the Induced Phonological Register. This is a test that evaluates the speech of boys and girls from a qualitative point of view through the spontaneous naming or repetition of words. Likewise, it qualitatively compares the production of the subject to the average of a group of children of his or her age. This record consists of 57 cards with images that cover the fundamental phonological spectrum of Spanish. In its application, sheets of drawings are shown where the child must say what he or she sees, repeating if a mistake is made [55]. There are no psychometric data for this test, although its use has been substantiated in the Hispanic population [56].

On the other hand, Laura Bosch’s phonological evaluation test was used [57]. This test allows the child’s phonological development to be determined, as well as the simplification processes and speech patterns of the subject. It is a test that is applied through induced language, in which the boy or girl has to answer questions related to 12 sheets that represent everyday situations. It contains a total of 62 phonemic elements with 32 words that the subject must say. In its application, sheets of drawings are shown in which images and actions are seen that include words that the subject must pronounce. The number of errors made and the erroneous phonemes are recorded [39]. This test offers reliability data between 0.98 and 0.99.

Both tests are designed for the child to produce words that contain all the phonemes corresponding to the Spanish phonological system through visual stimuli and presented drawings. They are the most commonly used standardized tests in Spanish to assess the phonetic–phonological area. In the first case, this is performed through naming and repetition tasks, while in the second test, it is carried out through directed language. In this way, the articulatory characteristics of the study participants can be observed.

## 3. Results

The results obtained show that people with 22q11.2 Syndrome experience alterations in the acquisition of speech sounds, producing the results set out below.

Regarding the results of the test called Induced Phonological Register [54], a series of tables and graphs are obtained referring to the number of errors and the different types of phonemic–phonological errors that people with this syndrome exhibit. The authors of the test consider that each phonemic mistake, therefore, constitutes an error in the production of the Word (wrong Word). Each mistake or error occurs when a phoneme is articulated incorrectly. It is related to its pronunciation but not to its lexical meaning (Table 2).

It has been confirmed that all the boys and girls evaluated made errors in the test called Induced Phonological Register, with an average of 15.25 wrong words for each child and 20.62 wrong phonemes. However, it should be noted that subject 8 has increased the mean of errors made both in words and in phonemes, thus reflecting the variability of the syndrome. The percentages represent the number of participants with errors or mistakes in the tasks.

The following table with its respective graphs is included to analyze the different types of phonemic–phonological errors. The graphs display both the total percentage of each of the different phonemic–phonological errors (incorrectly articulated phonemes) as well as the most frequent types of errors that each subject produces (Table 3).

Specifically, regarding the types of errors, most (73%) of the phonemic–phonological errors made correspond to omission processes. In this case, the children omitted the production of the phoneme proposed by the evaluation test. Among the most frequent errors are also distortion errors, occupying 16% of the total. Addition errors occur in 8% of cases and, lastly, distortion processes appear in 3% of cases.

**Figure 1:**
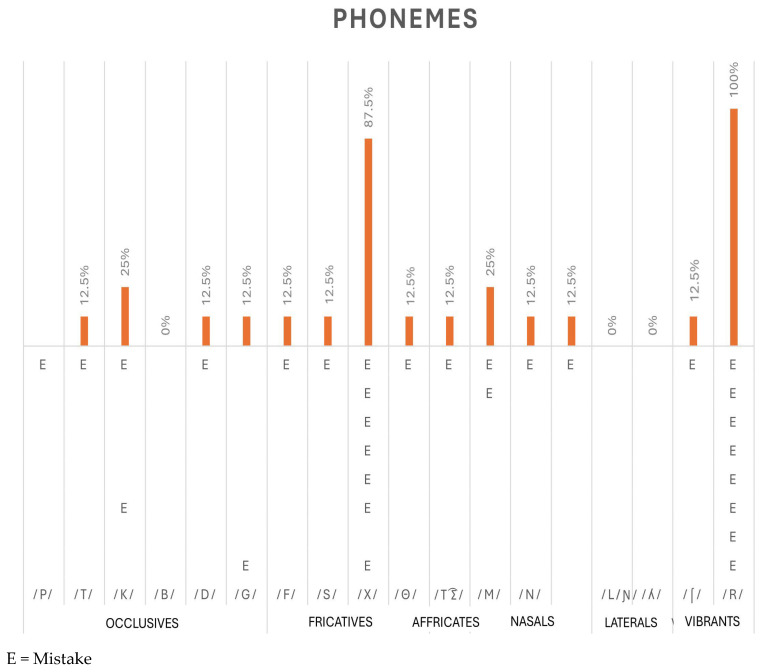
Percentage of wrong phonemes BW.

Regarding the results of the Laura Bosch Phonological Register test [39], some complementary data to the previous data have been obtained. For example, regarding the production of phonemes, taking into account their mode of articulation, especially in relation to the phonemes in which the person with 22q11.2 has more difficulty with respect to their production. In Figure 1, multiple difficulties can be observed in different speech sounds, specifically in fricatives and vibrants, where 100% of subjects make different types of phonemic errors in the phoneme /r/ and 87.5% in the phoneme /x/. Consequently, the stop /k/ and the nasal /m/ are also altered in 25% of the users in the study. To a lesser extent, 12.5% of the subjects reported problems emitting the stop phonemes /p, /t/, d/, /g/, the fricative phonemes /f/, /s/, /θ/, the affricate phoneme /tʃ/, the nasal phonemes /n/ and /ɲ/, and the vibrant /∫/. Lastly, there are no errors in the stop phoneme /b/, nor in the lateral phonemes /ʎ/ and /ʎ/. Note: The letter E represents the error of each of the participants.

**Figure 2:**
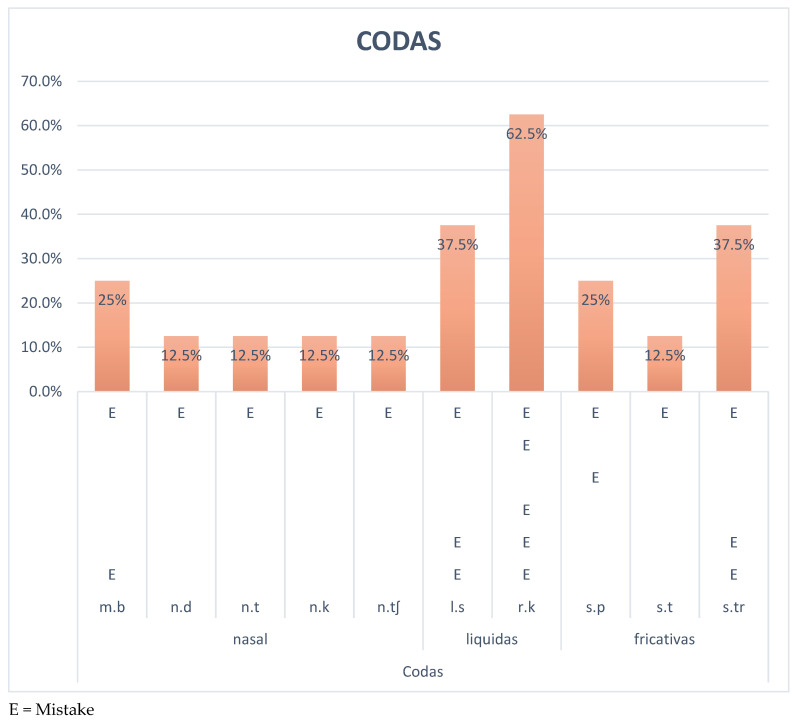
Percentage of wrong codas BW.

Regarding the second group of elements that are analyzed, syllabic codas, a series of results are obtained and presented in Figure 2. The coda that presents the greatest difficulties for users is undoubtedly the liquid /r.k/ with 62.5% mistakes. This percentage is followed by the liquid /l.s/ and the fricative /s.tr/, where 37.5% of users experience difficulties. Similarly, the nasal coda /m.b/ and the fricative /s.p/ were not acquired by the users of the study, giving rise to 25% mistakes. In the end, the nasal codas /n.d/, /n.t/, /n.k/, and /n.tʃ/ and the fricative coda /s.t/ registered 12.5% mistakes. Note: The letter E represents the error of each of the participants.

Concerning consonant clusters with /r/, we find that half of the users have difficulties in producing the clusters /gr/ and /br/, followed by the clusters /tr/ and /fr/ with 37.5% mistakes. Consequently, 25% of the subjects in this study had problems producing the consonant clusters /dr/ and /kr/, with the /pr/ cluster being the least affected, with only 12.5% mistakes.

Concerning the consonant clusters with /l/, we can observe that the most affected, by far, is in the /kl/ cluster, where 37.5% of subjects made errors in their utterances. As for the rest of the consonant clusters with /l/, /pl/, /gl/, /bl/, /fl/, there was only one user, representing 25.5%, who had not yet acquired it.

Now, if we take into account the consonant clusters with /r/ and /l/ as a whole, it can be seen that the clusters where the users exhibit the greatest difficulties are those that contain the phonemes /b/ and /g/ with 50% mistakes. They are followed by those consonant groups with the sounds /t/, /k/, and /f/, where 37.5% of the subjects exhibit difficulties, respectively. Lastly, with 25% mistakes, the consonant cluster with the sound /d/ and the cluster with the sound /p/ with 12.5% mistakes.

Finally, regarding the diphthongs, the study subjects show anomalies in all of them. But, it is in the diphthong /au/ where half (50%) experience greater difficulties. Then, the most affected syllabic nucleus is the diphthong /ei/, with an error rate of 35.5%. The diphthongs /ue/ and /ie/ and /io/ register an error rate, respectively, of 25% and 12.5%.

## 4. Discussion

The results obtained in this study show alterations in the speech and language of boys and girls with 22q11.2 deletion syndrome, especially concerning the phonemic–phonological characteristics considered the goal of this research. To date, there are only two studies that analyze these characteristics in the Spanish population. There are several conducted on speakers of English as a mother tongue, but it must be taken into account that these are two different languages, with a very different phonemic–phonological system.

Taking into account the parameters of normality of phoneme acquisition according to Bosch, all the subjects exhibit phonemic–phonological development lower than that expected for their age [39]. However, our results are consistent with research previously carried out by authors such as [40,44], who found that subjects with 22q11.2 deletion syndrome have greater difficulties in the production of vibrant, fricative, and liquid phonemes and the consonant clusters /r/. In contrast to our results, in which only one user presented difficulties in the affricate /tʃ/, D’Antonio et al. [42] and the studies by Sebastián- Lázaro, Brun-Gasca, and Fornieles [40,44] agree that this sound is one of the phonemes where people with 22q11.2 deletion syndrome experience greater difficulties. However, in the studies by D’Antonio et al. [42], the subjects had difficulties with nasal sounds, consistent with ours.

In our study, it should be noted that none of the participants older than 8 years had difficulties producing the phonemes /p/, /t/, /b/, /d/, /f/, /s/, /θ/, /tʃ/, /l/, /n/, highlighting the difficulties in producing the phonemes /k/, /r/, and consonant clusters. These results are linked to the study carried out by Sebastián-Lázaro, Brun-Gasca, and Fornieles [44], except for the altered phonemes /s/ and /x/ in our research. As in previous research, only the speech of a five-year-old subject was intelligible in our study.

At the same time, if we focus on consonant clusters, our study follows the line of the same previous study, where a late acquisition of these clusters was observed, and people with 22q11.2 deletion syndrome had greater difficulty in the diction of consonant + /r/ than of consonant + /l/.

Concerning the number of erroneous phonemes, it should be noted that the participants in our study emit an average of 20,625 erroneous phonemes. These data are comparable with the recent research by Sebastián-Lázaro, Brun-Gasca, and Fornieles [44], where the subjects emit an average of 19.3 erroneous phonemes.

Regarding the use of compensatory sounds, none of our participants made use of these, and they made phonemic errors referring to omissions, substitutions, additions, and distortions on all occasions, in line with the previous study. In our research, the most frequent errors of articulation of speech sounds were omissions, in line with the research carried out by Bosch-Galcerán [39]. However, previous studies, such as those of Havkin, Tatum III, and Shprintzen [58]; D’Antonio et al. [42]; and Scherer et al. [47], had already recorded the use of these compensatory sounds to replace the production of other sounds during childhood.

Likewise, the participants in this study exhibited difficulties in correctly emitting several phonemes. This result is consistent with the findings of Golding-Kushner and Shprintzen [41], who state that the majority of children with 22q11.2 show defective articulation due to the use of altered phonemes. The results of the present study show that articulatory precision is diminished because the points of articulation of the phonemes are not marked. These results concur with those of Solot et al. [14], who observed that, when producing phonemes, children with 22q11.2 perform with lower precision.

The specific difficulty in articulating these phonemes is in line with other studies. Speech sound disorders characterized by developmental errors are often observed [26,59]. Obligatory features of velopharyngeal and palatal insufficiency are common and include weak pressure consonants, nasalization of phonemes, and audible nasal air emission during the production of oral pressure consonants. Children frequently demonstrate compensatory misarticulations [26,42,59]. Glottal stops, pharyngeal fricatives/stops, nasal fricatives, laryngeal fricatives, and, less frequently, clicks are observed. However, it is important to note that, to date, despite several studies addressing velopharyngeal insufficiency in the context of 22q11.2 Syndrome, there is no scientific evidence regarding how this characteristic directly affects phoneme production specifically in 22q11.2 Syndrome, and existing hypotheses are still considered simplistic [60].

Thus, the results indicate a more delayed and later acquisition of the phonological and phonemic area, in agreement with Scherer et al. [47] and Golding-Kushner et al. [41], who report that the speech and language development of children with 22q11.2 deletion syndrome differs from that of boys and girls without the syndrome. All of this concurs with Solot et al. [26] and Sebastián-Lázaro, Brun-Gasca, and Fornieles [40,44], who state that people with this syndrome acquire language milestones with a delay and have a greater tendency to develop phonological alterations. However, the participants in our study follow a speech and language development that is consistent with the typical developmental pattern, in accordance with the research carried out by Sebastián-Lázaro, Brun-Gasca, and Fornieles [40,44].

According to the data presented, we can conclude that subjects with 22q11.2 Syndrome report greater difficulties in the production of late acquisition phonemes such as vibrant rhotics, fricatives, stops, nasals, consonant clusters with /r/, r.k syllabic codas and the diphthong /ue/, corroborating the initial hypothesis on the one hand and disproving it on the other since, in our study, the subjects did not use compensatory sounds. Consequently, they are susceptible to speech therapy intervention, given that the improvement of linguistic skills will affect adequate phonemic–phonological development. At this point, it should be emphasized that the objective of this research was to describe the phonetic–phonological characteristics of this population, which has been developed in the present article. However, it should not be forgotten that some of these difficulties may be due to the anatomical–physiological characteristics inherent to the syndrome (such as velopharyngeal insufficiency), but due to the great heterogeneity of the population, it opens up possible avenues for future research. Therefore, the main hypothesis of the study is fulfilled since it has been verified that people with 22q11.2 Syndrome present certain difficulties in their phonemic and phonological development, with some errors that did not correspond to the equivalent of their chronological age.

Difficulties in the pronunciation of the phonemes specific to each language and the intelligibility of speech can also negatively affect the interaction of people with 22q11.2 Syndrome with their families, which has an impact on language production [53,61,62]. Therefore, we must not forget that it is necessary to continue insisting on the need to specifically work on the phonetic–phonological area throughout all stages of development.

As possible limitations of the study, we can highlight that the sample could have been larger. It is true that the age of the participants and the range between them makes it difficult to generalize the results, but currently, 22q11.2 Syndrome is included in the category of rare diseases, so the sample is reduced in these investigations. On the other hand, the results should focus primarily on the Spanish language, but we cannot affirm that these characteristics are the same in other languages. Lastly, it is important to acknowledge that although our results have shown additional evidence regarding the phonemic–phological profile in 22q11.2, the profound nature of these alterations remains to be determined. We also consider that it would be interesting to replicate this study in person in a complementary way.

## 5. Conclusions

This study has shown, once again, that children with 22q11.2 Syndrome have a higher risk of developing sociocommunicative problems in the future [4,63,64]. In conclusion and in line with the research by Solot et al. [26], the linguistic profile of people with 22q11.2 Syndrome should be reviewed by studies with larger samples due to the high heterogeneity of their characteristics. It is, therefore, very important to include them in language intervention programs from the moment the diagnosis is established [49]. Therefore, it is considered essential to increase and complete the scientific evidence in this regard with new research that might allow us to support the starting point of speech therapy intervention.

## Figures and Tables

**Table 1:** Characteristics of the sample.

Subject	Chronological Age	Gender	Educational Level in School
Subject 1	5.91	Male	1° Grade Primary School
Subject 2	11.08	Female	5° Grade Primary School
Subject 3	7.16	Female	2° Grade Primary School
Subject 4	6.66	Male	1° Grade Primary School
Subject 5	13.66	Female	2° Grade Secondary School
Subject 6	14.41	Male	3° Grade Secondary School
Subject 7	10.5	Female	4° Grade Primary School
Subject 8	8.58	Male	3° Grade Primary School

**Table 2:** Number of wrong phonemes and words in the induced phonological register.

Subject	Total Number of Wrong Words	Total Number of Wrong Phonemes
Subject 1	17	18
Subject 2	4	4
Subject 3	18	22
Subject 4	10	10
Subject 5	8	7
Subject 6	4	4
Subject 7	10	11
Subject 8	51	89
Total	122	165
AVERAGE	15.25	20.62

**Table 3:** Types of phonetic–phonological errors according to the subjects.

Subject	Number of Omissions	Number of Substitutions	Number of Additions	Number of Distortions
Subject 1	9	2	1	5
Subject 2	2	0	0	2
Subject 3	15	2	0	4
Subject 4	6	0	0	4
Subject 5	1	3	0	4
Subject 6	1	0	0	4
Subject 7	1	3	3	4
Subject 8	86	3	0	0
Total	121	13	4	27
AVERAGE	15.12	1.62	0.5	3.37

## Data Availability

The raw data supporting the conclusions of this article will be made available by the authors without undue reservation.
